# Relationship of Physical Activity With Anxiety and Depression Symptoms in Chinese College Students During the COVID-19 Outbreak

**DOI:** 10.3389/fpsyg.2020.582436

**Published:** 2020-11-20

**Authors:** Ming-Qiang Xiang, Xian-Ming Tan, Jian Sun, Hai-Yan Yang, Xue-Ping Zhao, Lei Liu, Xiao-Hui Hou, Min Hu

**Affiliations:** ^1^School of Sport and Health, Guangzhou Sport University, Guangzhou, China; ^2^Scientific Laboratory Center, Guangzhou Sport University, Guangzhou, China; ^3^School of Athletic Training, Guangzhou Sport University, Guangzhou, China; ^4^Graduate School, Guangzhou Sport University, Guangzhou, China; ^5^School of Nursing, Liaoning University of Traditional Chinese Medicine, Shenyang, China

**Keywords:** COVID-19, physical activity, anxiety, depression, college students

## Abstract

**Introduction:**

During the COVID-19 outbreak, many citizens were asked to stay at home in self-quarantine, which can pose a significant challenge with respect to remaining physically active and maintaining mental health. This study aimed to evaluate the prevalence of inadequate physical activity, anxiety, and depression and to explore the relationship of physical activity with anxiety and depression symptoms among Chinese college students during quarantine.

**Method:**

Using a web-based cross-sectional survey, we collected data from 1,396 Chinese college students. Anxiety and depression were assessed with the Self-Rating Anxiety Scale (SAS) and the Self-Rating Depression Scale (SDS), respectively. The data on physical activity were collected by types of physical activity and the International Physical Activity Questionnaire (IPAQ-SF).

**Results:**

During the COVID-19 outbreak, about 52.3% of Chinese college students had inadequate physical activity. The rates of anxiety and depression symptoms were 31.0 and 41.8%, respectively. A high level of physical activity (β = −0.121, *P* < 0.001) was significantly closely associated with low anxiety, while a moderate (β = −0.095, *P* = 0.001), or high (β = −0.179, *P* < 0.001) level of physical activity was significantly closely associated with reduced depression after adjusting confounding demographic factors. Moreover, specific types of physical activity, such as stretching and resistance training, were negatively correlated with both anxiety and depression; doing household chores was negatively correlated with depression.

**Conclusion:**

Our findings highlight specific levels and types of home-based physical activities that need to be taken into consideration to protect the mental health of college students during the COVID-19 epidemic.

## Introduction

The coronavirus disease 2019 (COVID-19) outbreak began in Wuhan (Hubei Province, China) in December 2019. It then spread first in China and soon afterward throughout the world. The World Health Organization (World Health Organisation, 2020a) reported that as of July 9, 2020, more than 12 million cases had been confirmed in more than 200 countries and regions. The large-scale epidemic has brought not only the risk of death from the viral infection but also public mental health problems, such as anxiety and depression symptoms, worldwide (Elbay et al., 2020; Huang and Zhao, 2020; Rajkumar, 2020; Vindegaard and Eriksen Benros, 2020). To contain the diffusion of infection, Chinese governments have implemented unprecedented and effective quarantine measures and delayed starting schools. Many healthy college students are being asked to stay at home in self-quarantine, while implementing the emergency policy of “suspending classes without stopping learning.” This means that tens of million Chinese college students are facing challenges in navigating online learning while coping with the stresses of daily life, which are expected to bring a mental health burden. For example, recent studies showed that the prevalence rates of anxiety and depression symptoms were around 24.9 and 9.0%, respectively, among Chinese college students during the COVID-19 outbreak (Cao et al., 2020; Tang et al., 2020). Therefore, psychiatrists and researchers should be aware of these mental health problems and their correlates and should develop measures and implement interventions appropriate for this situation (Liu et al., 2020; Rajkumar, 2020).

Staying at home for prolonged periods of time can also increase sedentary behavior and decrease the levels of physical activity, and may lead to a mental health burden (Hemphill et al., 2020). However, home-based physical activity might be a valuable tool to help individuals remain calm and continue to protect their mental health during home-quarantine. For example, Chen et al. (2020) pointed out that staying active and maintaining regular physical activity may help students recuperate from the mental health problems they experienced while in quarantine during the COVID-19 crisis. Furthermore, WHO has released guidance intended for people in self-quarantine, including practical advice on how to stay physically active and reduce sedentary behavior while at home (World Health Organisation, 2020b). However, previous studies have focused mainly on evaluation of the mental health of college students and the risk factors of anxiety and depression during quarantine (Cao et al., 2020; Odriozola-González et al., 2020; Tang et al., 2020). Little is known about the details of home-based physical activity and its association with reduced anxiety/depression symptoms.

To address these key evidence gaps, in this study, we evaluate the anxiety and depression of Chinese college students during the COVID-19 outbreak and explore the relationship of physical activity with anxiety and depression symptoms. We hypothesized that a certain percentage of college students had inadequate physical activity and would experience depression and anxiety during the epidemic, while special types and levels of physical activity would be associated with reduced anxiety/depressive symptoms.

## Materials and Methods

### Participants and Procedure

The target participants comprised Chinese graduate and undergraduate students who were staying at home in self-quarantine and engaging in online learning during the COVID-19 outbreak. We conducted a cross-sectional anonymous web-based survey to collect data from February 25 to March 5, 2020. The questionnaire survey was imported to the web survey platform Chinese Survey Star^1^ (Changsha Ranxing Science and Technology, Shanghai, China) and distributed on the WeChat public platform using a snowball sampling strategy. A total of 30 university students and 10 teachers from our university were selected as “original deliverers.” They sent the questionnaire links to WeChat groups of college students. To recruit more participants, the questionnaire links were also distributed among WeChat groups of the respondents’ classmates. Chinese college students were able to access the survey using WeChat and fill out the questionnaire anonymously by clicking the link or scanning the QR code. After participants reach the survey homepage, an online consent form is displayed, before the users see the questionnaire. If the participants have no objection to the survey objective in the consent form, they officially start the survey by clicking the “Next” button, or they exercise their right to cease the survey. This web-based questionnaire was completely voluntary and non-commercial.

A total of 2,343 students from 76 college student WeChat groups were invited to participate in the survey. Finally, 1,421 college students completed the survey, a response rate of 60.6%. Before data processing, we applied a series of exclusion criteria: (1) no clear name given for the college and major; (2) any obvious discrepancy between the level of education (undergraduate or graduate) and school academic system; (3) the same kind of response given to all questions. According to these exclusion criteria, 1,396 questionnaires were considered valid for inclusion in the analysis.

### Ethics Statement

This study was approved by the Human Experimental Ethics Board of Guangzhou Sport University (Approval no. 2020LCLL-004) in accordance with the Declaration of Helsinki. Each participant provided electronic informed consent before seeing the questionnaire. Participants could withdraw from the survey at any moment without providing any justification.

### Measurements

Demographic variables included gender (male or female), age, only child (yes or no), single parent (yes or no), education (undergraduate or graduate), and the number of times focusing on COVID-19 information. The focus on COVID-19 information was measured by the filling in the number of times per day spent on browsing information relating to the COVID-19 pandemic over the previous week. The type of physical activity, physical activity level, anxiety, and depression were assessed as follows.

#### Physical Activity Level

The physical activity level was measured using the short form of the International Physical Activity Questionnaire (IPAQ-SF), which has been validated and recommended as an efficient method to assess physical activity (Craig et al., 2003). Participants reported the frequency and duration of their vigorous and moderate physical activities and walking per week. The metabolic equivalent of task (MET) of vigorous activity is 8.0, that of moderate activity is 4.0, and that of walking is 3.3. According to the official guideline criteria (Sjostrom, 2020) and previous studies (Kim et al., 2019), the participants’ physical activity level could be categorized as high, moderate, or low as follows.

(1) Category high: The pattern of activity was classified as high if it met either of the following criteria: (a) vigorous activity at least 3 days in a week achieving a minimum of 1500 MET-min/week, or (b) 7 or more days of any combination of walking, moderate-intensity, or vigorous-intensity activities achieving a minimum of 3000 MET-min/week.

(2) Category moderate: The pattern of activity was classified as moderate if it met any one of the following three criteria: (a) 3 or more days of vigorous activity of at least 20 min per day in a week, or (b) 5 or more days of moderate-intensity activity and/or walking of at least 30 min per day in a week, or (c) 5 or more days of any combination of walking, moderate-intensity, or vigorous activities achieving a minimum of at least 600 MET-min/week.

(3) Category low: Those individuals who did not meet criteria for Categories 1 or 2 were considered to have a low physical activity level.

Additionally, the prevalence of inadequate physical activity was also calculated according to current WHO guidelines (2020b), which recommended that adults engage in at least 75 min of vigorous-intensity physical activity per week, at least 150 min of moderate physical activity, or any equivalent combination of the two.

#### Type of Physical Activity

Before conducting the survey, 10 college students and 3 physical education teachers were invited for an interview discussing the most frequent types of physical activity for college students during home quarantine. Finally, we selected 10 common home-quarantine physical activities, including household chores, walking, jumping, running, stretching, resistance training, bodybuilding, yoga, Tai chi, and sports (e.g., football, basketball, volleyball). If an activity was not on the list, it was categorized as “other.” Participants were asked: “During past week, in which of the following home-quarantine physical activities or exercises did you participate for at least 15 min (multiple choice)?” The questionnaire included 10 items on specific home-quarantine physical activities and 1 “other” physical activity. Though the activity type “other” is presented, the results are not discussed further because of the mix of different activity types in that category.

#### Anxiety and Depression

Zung’s Self-Rating Anxiety Scale (SAS) and Self-Rating Depression Scale (SDS) were used to assess anxiety and depression symptoms, respectively (Zung, 1965, 1971). Both the SAS and SDS are composed of 20 items, each of which is assessed using a 4-point Likert scale ranging from 1 (*not at all or a little of the time*) to 4 (*most of the time or all the time*). Higher total scores indicate greater anxiety or depression. The raw score was standardized using the following formula: standard score = INT (1.25 × raw score). A standard scores exceeding 50 indicates that the individual suffers from anxiety or depression symptoms (Zung, 1973; Wang, 1984). Both the SAS and SDS have been demonstrated to have acceptable validity and reliability in the Chinese population (Lee et al., 1994; Tao and Gao, 1994) and have been applied in clinical practice and research for the evaluation of anxiety and depression related to the COVID-19 epidemic (Guo et al., 2020; Liang et al., 2020; Ma et al., 2020).

### Statistical Analysis

All of the statistical analyses were performed with SPSS version 23.0 (IBM Corp., Armonk, NY, United States). According to the research purpose, measurement data were expressed as the mean ± standard deviation (SD), while independent sample *t*-tests were used for group comparisons. In the multiple group comparisons, one-way analysis of variance was used. Pearson’s correlation was used to examine the association between depression, anxiety, age, and the number of times focusing on COVID-19. Additionally, linear regressions were applied to analyze the relationship of physical activity levels and types with anxiety and depression symptoms. Anxiety and depression scores were used as dependent variables, while physical activity level and type were used as an independent variable. Physical activity level was recoded as two dummy variables (reference group = low level): moderate and high level. Significance levels were set at 0.05, and all tests were two-sided.

## Results

### Participants’ Characteristics

A total of 1,396 participant surveys were used in the analysis, including 881 (63.1%) males and 515 (36.9%) females. The mean (standard deviation) age of the participants was 20.68 (1.84) years. Among these samples, 1,314 (94.1%) of participants were undergraduate students, 427 (30.6%) of participants were the only child in the family, and 126 (9.0%) lived in a single-parent family. The mean (standard deviation) number of times focusing on COVID-19 was 3.94 (2.80) per day. Regarding physical activity, the mean (standard deviation) time spent on vigorous activity, moderate activity, and walking was 90.09 (78.53), 133.34 (79.70), and 157.45 (95.31) min/week, respectively. However, 730 (52.3%) of Chinese college students had inadequate physical activity. The overall prevalence rates of anxiety and depressive symptoms were 31.0 and 41.8%, respectively (Table 1).

**TABLE 1:** Characteristics of college students in the sample.

Variable	Total (*n* = 1396)
Age (years), Mean ± SD	20.68 ± 1.84
Gender, *n*(%)	
Male	881 (63.1)
Female	515 (36.9)
Only child, *n*(%)	
No	969 (69.4)
Yes	427 (30.6)
Single parent, *n*(%)	
No	1270 (91.0)
Yes	126 (9.0)
Education, *n*(%)	
Undergraduate	1314 (94.1)
Graduate	82 (5.9)
The number of times focusing on COVID-19(*n*), Mean ± SD	3.94 ± 2.80
Physical activity (min/week), Mean ± SD	
Vigorous activity	90.09 ± 78.53
Moderate activity	133.34 ± 79.70
Walking	157.45 ± 95.31
Insufficient physical activity (%)	730 (52.3%)
Anxiety Symptoms, *n* (%)	
Yes	433 (31.0)
No	963 (69.0)
Depression symptoms, *n* (%)	
Yes	583 (41.8)
No	813 (58.2)

Analysis of demographic characteristics of anxiety and depression symptoms is displayed in Table 2. Female college students exerted higher SAS scores than male college students (*P* = 0.023). College students who lived in an only child family reported significantly higher SAS scores than those who lived in a non-only child family (*P* < 0.001). Regarding depression symptoms, female college students exerted higher SDS scores than male college students (*P* = 0.009). Undergraduate students reported significantly higher SDS scores than graduate students (*P* = 0.002).

**TABLE 2:** Demographic characteristics influencing anxiety and depression symptoms (mean ± SD).

	Anxiety	*r/t*	*P*	Depression	*r/t*	*P*
Age		0.001	0.984		−0.008	0.761
Gender		**−2.28**	**0.023**		0.071	0.943
Male	35.11 ± 8.33			37.20 ± 10.04		
Female	36.16 ± 8.30			37.17 ± 8.97		
Only child		**−3.98**	**< 0.001**		**−2.63**	**0.009**
Yes	34.16 ± 8.20			36.17 ± 9.47		
No	36.08 ± 8.33			37.64 ± 9.71		
Single parent		0.335	0.738		−0.743	0.458
Yes	35.73 ± 7.748			36.58 ± 9.458		
No	35.47 ± 8.391			37.25 ± 9.678		
Education		1.40	0.162		**3.06**	**0.002**
Undergraduate	35.57 ± 8.39			37.39 ± 9.66		
Graduate	34.24 ± 7.27			34.04 ± 9.09		
The number of times focusing on COVID-19		0.018	0.510		−0.031	0.248

*Bold values indicate statistical significance at *p* < 0.05.*

### Physical Activity Level Factor of Mental Health During COVID-19 Outbreak

Significant differences in anxiety and depression were observed among the three physical activity levels in the comparative analysis (Supplementary Table S1).

The linear regression analyses showed that participants with a high level (β = −0.121, *P* < 0.001) of physical activity were significantly associated with low anxiety, while a moderate (β = −0.095, *P* = 0.001) or high (β = −0.179, *P* < 0.001) level of physical activity was significantly associated with reduced depression after adjusting confounding demographic factors (Table 3).

**TABLE 3:** Linear regression analyses of the relationships of physical activity level with anxiety and depression symptoms.

	Anxiety				Depression			
		
Independent variable^a^	β	*T*	*P*	95%CI	β	*t*	*P*	95%CI
Low level (reference)								
Moderate level	−0.012	−0.391	0.695	−1.622 to 1.082	−0.095	−3.221	**0.001**	−4.096 to −0.995
High level	−0.121	−4.066	**<0.001**	−3.966 to−1.385	−0.179	−6.071	**< 0.001**	−6.061 to −3.101

*^*a*^Control gender, age, only child, single parent, education and the number of times focusing on information about COVID-19 per day. Bold values indicate statistical significance at *p* < 0.05.*

### Physical Activity Type Factor of Mental Health During the COVID-19 Outbreak

Significant differences in anxiety were observed for two types of physical activities, namely, stretching and resistance training (*P* < 0.01). Meanwhile, significant differences in depression were found for three types of physical activities: household chores, stretching, and resistance training (*P* < 0.001) (Supplementary Table S2).

In the linear regression analyses (Table 4), stretching (β = −0.082, *P* = 0.005) and resistance training (β = −0.058, *P* = 0.042) were significantly associated with low anxiety, while household chores (β = −0.120, *P* < 0.001), stretching (β = −0.122, *P* < 0.001), and resistance training (β = −0.131, *P* < 0.001) were significantly associated with reduced depression after adjusting for confounding demographic factors. On closer inspection, household chores were associated with the lowest depression compared with other types of physical activity.

**TABLE 4:** Linear regression analyses of the relationships of physical activity type with anxiety and depression symptoms.

	Anxiety				Depression			
		
Independent variable^*a*^	β	*t*	*P*	95%CI	β	*t*	*P*	95%CI
Walking	0.013	0.460	0.645	−0.889 to 1.434	0.017	0.621	0.535	−0.899 to 1.732
Household chores	−0.047	−1.669	0.095	−2.162 to 0.174	−0.120	−4.323	**< 0.001**	−4.238 to−1.593
Jumping	−0.004	−0.135	0.893	−1.537 to 1.339	0.023	0.781	0.435	−0.980 to 2.277
Yoga	0.008	0.270	0.788	−1.583 to 2.087	0.025	0.867	0.386	−1.159 to 2.996
Tai chi	0.012	0.449	0.653	−1.973 to 3.145	0.006	0.226	0.822	−2.564 to 3.231
Bodybuilding	0.037	1.303	0.193	−0.597 to 2.957	0.048	1.702	0.089	−0.267 to 3.758
Running	−0.034	−1.243	0.214	−2.271 to 0.509	−0.031	−1.175	0.240	−2.517 to 0.631
Stretching	−0.082	−2.788	**0.005**	−2.949 to −0.513	−0.122	−4.237	**< 0.001**	−4.357 to 1.599
Sports	−0.029	−1.083	0.279	−1.998 to 0.577	−0.017	−0.633	0.527	−1.929 to 0.987
Resistance training	−0.058	−2.034	**0.042**	−2.512 to −0.046	−0.131	−4.650	**< 0.001**	−4.706 to −1.914
Other activities	−0.026	−0.946	0.344	−2.224 to 0.777	−0.004	−0.146	0.884	−1.825 to 1.572

*^*a*^Control gender, age, only child, single parent, education and the number of times focusing on information about COVID-19 per day. Bold values indicate statistical significance at *p* < 0.05.*

## Discussion

The COVID-19 epidemic has been spreading worldwide, and many citizens have been asked to stay at home in self-quarantine, which is associated with reduced physical activity and increased mental health burden (Hemphill et al., 2020). This study aimed to evaluate the anxiety and depression of college students during the COVID-19 outbreak in China and explore the relationship of anxiety and depression symptoms with physical activity types and levels.

Our findings indicated that the prevalence rates of anxiety and depression in college students were 31.0 and 41.8%, respectively. The results were both higher than the rates (24.9% for anxiety and for 9% depression) found in prior surveys among Chinese college students (Cao et al., 2020; Tang et al., 2020) but within the range (22.6–36.3% for anxiety and 16.5–48.3% for depression) among the general population in China during the same period reported by meta-analysis (Pappa et al., 2020). The inconsistent results may be related to the different psychological scales used and different survey times. First, our survey used the SAS/SDS to measure anxiety/depression symptoms, while other surveys used the Generalized Anxiety Disorder Scale (Cao et al., 2020) and Patient Health Questionnaire-9 (Tang et al., 2020). Thus, the different scales and cut-off scores applied by each survey could yield different results. Second, our survey was conducted in late February and early March, when the government had ordered a nationwide school closure and suggested “suspending classes without stopping learning” from late February 2020. Hence, the challenges for college students came not only from the effect of the virus on their life but also from the use of the new mode of online learning, which may increase anxiety or depression. Our findings also indicated that college students’ anxiety regarding the epidemic was associated with whether they were an only child, while depression during the epidemic was associated with gender, age, education, and only-child status.

This study showed about 52.3% of Chinese college students engaged in inadequate physical activity during the COVID-19 outbreak, nearly more than twice the global prevalence of inadequate physical activity (27.5%) under non-outbreak conditions (Guthold et al., 2018). Novel to our study were our findings of a meaningful association between physical activity level and mental health in Chinese college students during the COVID-19 outbreak, even after adjusting confounding demographic factors, such as gender, age, family background, education level, and the number of times a day focusing on information about COVID-19. College students who engaged in a high level of physical activity had lower anxiety than those who engaged in low levels of physical activity, while individuals who engaged in moderate and high levels of exercise had lower depression than those with a low level of physical activity. These results indicated that the association between physical activity and depression was greater than that between physical activity and anxiety during the COVID-19 crisis, which is consistent with a prior meta-analysis study, indicating that physical activity reduced depression with a medium effect (SMD = −0.5) and anxiety with a small effect (SMD = −0.38) (Rebar et al., 2015). It is plausible that the relationships we found are causal because they are consistent with randomized controlled trials indicating the beneficial effects of physical activity on anxiety and depression (Carek et al., 2011; Rebar et al., 2015), as well as dose-response studies, suggesting that more activity has a greater beneficial effect on mental health (Legrand and Heuze, 2007; Wipfli et al., 2008). Physical activity might reduce anxiety/depressive symptoms through a variety of psychosocial and biological mechanisms, such as increasing neurotrophic factor (BDNF) and endogenous opioids (endorphins), improving the immune system, or promoting self-esteem (Balchin et al., 2016). Thus, a moderate or high level of physical activity can attenuate the symptoms and consequences of quarantine-induced anxiety/depression through complex and powerful systemic neuroprotective effects.

Regarding physical activity type, our survey showed that among 10 types of physical activity, 2 types of physical activity (stretching and resistance training) were associated with lower anxiety, and 3 types of physical activity (household chores, stretching, and resistance training) were associated with lower depression. This result is inconsistent with a prior survey finding that all of the physical activity types, including social and non-social forms, were associated with lower mental health burden among 1.2 million individuals (Chekroud et al., 2018). The lack of association between all types of physical activity and anxiety/depression symptoms might be related to the fact that college students’ activity levels declined during the COVID-19 outbreak. Despite this, we found that college students doing household chores had the lowest depression. The reason might be that household chores, such as family members cooking together, like other social activities, promote resilience to stress and reduce depression (Chekroud et al., 2018). Thus, the prosocial benefits from household chores might contribute an additional benefit for mental health over other types of physical activity, especially during the COVID-19 outbreak. In addition, stretching and resistance training were conducive to reducing both anxiety and depression, which supports the “stay physically active during self-quarantine” recommendation by the Chinese Center for Disease Control and Prevention (2020) and World Health Organisation (2020b).

To sum up, it is important to implement strategies to further increase home-based physical activity when facing necessary social isolation or quarantine. The social networks, videos, and information search sites for health promotion among the general population could be applied to achieve the recommendations of the World Health Organisation (2020b), which suggest “150 min of moderate-intensity or 75 min of vigorous-intensity physical activity per week, or a combination of both.”

This study has several limitations: First, it is based on cross-sectional data; thus, causal relationships between physical activity and anxiety/depression symptoms should be interpreted with great caution. Future research may apply a longitudinal or experimental design to validate the causal relationships among these variables. Second, because of the limited resources available and the quarantine, the snowball sampling strategy and online self-report survey were adopted, which might be subject to participation bias, social desirability bias, and shared method variance. Future studies can improve representative samples of Chinese college students, and apply objective methods—such as ActiGraph accelerometers and assessment by mental health professionals—to assess physical activity and anxiety/depression disorder. Third, due to the sudden occurrence of the disaster, we were unable to compare the differences between college students’ physical activity levels and mental health before and after the COVID-19 outbreak. Furthermore, we only listed 10 common home-quarantine physical activities, which may limit the diversity and representativeness of physical activity type. Lastly, information about college students’ socioeconomic level, type and size of housing, and frequency and duration of each physical activity type was not reported.

## Conclusion

This study found that during the COVID-19 outbreak, about 52.3% of Chinese college students had inadequate physical activity. The prevalence rates of anxiety and depression in college students were 31.0 and 41.8%, respectively. Moderate and high levels of physical activity, as well as specific types of physical activity, such as household chores, stretching, and resistance training, were protective factors against anxiety or depression among the college students. The current study expands the literature on physical activity and mental health during the COVID-19 outbreak and points to the need to promote home-based physical activity to protect the physical and mental health of college students and the general population.

## Data Availability Statement

The raw data supporting the conclusions of this article will be made available by the authors, without undue reservation.

## Ethics Statement

The studies involving human participants were reviewed and approved by the Human Experimental Ethics Board of the Guangzhou Sport University. The patients/participants provided their written informed consent to participate in this study.

## Author Contributions

X-HH, MH, and M-QX designed the study and wrote the protocols. M-QX, X-MT, and JS designed and selected the scales. H-YY and X-PZ participated in the data collection. LL undertook the statistical analysis. M-QX wrote the manuscript, which all authors helped revise. All authors contributed to and approved the final manuscript.

## Conflict of Interest

The authors declare that the research was conducted in the absence of any commercial or financial relationships that could be construed as a potential conflict of interest.

## Footnotes

1 https://www.wjx.cn/
