# Supplementary material for: Relationship of Physical Activity With Anxiety and Depression Symptoms in Chinese College Students During the COVID-19 Outbreak
Source: Front Psychol. 2020 Nov 20;11:582436. doi: 10.3389/fpsyg.2020.582436 (PMC7714784; doi:10.3389/fpsyg.2020.582436)
Supplement: Supplementary file 1 [file Table_1.docx]

**Table S1 Comparative analysis of anxiety and depression total score stratified by physical activity level**

|  | *n* | Anxiety | *F* | *p* | Depression | *F* | *p* |
| --- | --- | --- | --- | --- | --- | --- | --- |
| **Physical activity level** |  |  | 9.404 | <0.001 |  | 18.571 | <0.001 |
| Low | 538 | 44.96 ± 10.52 |  |  | 48.34 ± 11.97 |  |  |
| Moderate | 390 | 44.79 ± 10.44 |  |  | 45.81 ± 11.73 |  |  |
| High | 468 | 42.34 ± 10.21 |  |  | 43.77 ± 11.99 |  |  |

**Table S2 Comparative analysis of anxiety and depression total score stratified by physical activity type**

|  | *n* | Anxiety | *t* | *p* | Depression | *t* | *p* |
| --- | --- | --- | --- | --- | --- | --- | --- |
| Walking |  |  | -0.48 | 0.629 |  | 0.02 | 0.983 |
| No | 835 | 43.92 ± 10.63 |  |  | 46.11 ± 12.17 |  |  |
| Yes | 561 | 44.20 ± 10.21 |  |  | 46.09 ± 11.90 |  |  |
| Household chores |  |  | 1.469 | 0.142 |  | **7.69** | **<0.001** |
| No | 603 | 44.51 ± 11.01 |  |  | 47.85 ± 12.36 |  |  |
| Yes | 793 | 43.68 ± 10.01 |  |  | 44.77 ± 11.66 |  |  |
| Jumping |  |  | 1.22 | 0.223 |  | 1.53 | 0.126 |
| No | 1078 | 44.22 ± 10.41 |  |  | 46.37 ± 12.13 |  |  |
| Yes | 318 | 43.41 ± 10.63 |  |  | 45.19 ± 11.78 |  |  |
| Yoga |  |  |  |  |  | 0.238 | 0.812 |
| No | 1231 | 44.02 ± 10.43 | -0.815 | 0.853 | 46.13 ± 12.07 |  |  |
| Yes | 165 | 44.18 ± 10.73 |  |  | 45.89 ± 12.01 |  |  |
| Tai chi |  |  | -0.364 | 0.716 |  | -0.177 | 0.84 |
| No | 1328 | 44.01 ± 10.46 |  |  | 46.09 ± 12.08 |  |  |
| Yes | 68 | 44.49 ± 10.53 |  |  | 46.35 ± 11.62 |  |  |
| Bodybuilding |  |  | -0.742 | 0.458 |  | -0.371 | 0.711 |
| No | 1221 | 43.96 ± 10.30 |  |  | 46.06 ± 11.97 |  |  |
| Yes | 175 | 44.58 ± 11.56 |  |  | 46.42 ± 12.65 |  |  |
| Running |  |  | 1.527 | 0.127 |  | 1.606 | 0.108 |
| No | 1117 | 44.25 ± 10.56 |  |  | 46.36 ± 12.07 |  |  |
| Yes | 279 | 43.18 ± 10.04 |  |  | 45.06 ± 11.98 |  |  |
| Stretching |  |  | **3.06** | **0.002** |  | **5.731** | **<0.001** |
| No | 818 | 44.75 ± 10.72 |  |  | 47.64 ± 12.18 |  |  |
| Yes | 578 | 43.02 ± 10.01 |  |  | 43.93 ± 11.54 |  |  |
| Sports |  |  | 1.285 | 0.199 |  | 0.649 | 0.516 |
| No | 1066 | 44.23 ± 10.43 |  |  | 46.22 ± 11.96 |  |  |
| Yes | 330 | 43.39 ± 10.55 |  |  | 45.72 ± 12.39 |  |  |
| Resistance training |  |  | **3.429** | **0.001** |  | **5.889** | **<0.001** |
| No | 907 | 44.74 ± 10.51 |  |  | 47.47 ± 12.04 |  |  |
| Yes | 489 | 42.73 ± 10.26 |  |  | 43.57 ± 11.67 |  |  |
| Other activities |  |  | 0.413 | 0.680 |  | -0.699 | 0.485 |
| No | 1166 | 44.09 ± 10.40 |  |  | 46.00 ± 12.04 |  |  |
| Yes | 230 | 43.77 ± 10.80 |  |  | 46.61 ± 12.17 |  |  |

Bold values indicate statistical significance at *p* < 0.05
